# Controllable Fabrication of Zn^2+^ Self-Doped TiO_2_ Tubular Nanocomposite for Highly Efficient Water Treatment

**DOI:** 10.3390/molecules28073072

**Published:** 2023-03-30

**Authors:** Hassan M. A. Hassan, Ibrahim H. Alsohaimi, Amr A. Essawy, Mohamed R. El-Aassar, Mohamed A. Betiha, Alhulw H. Alshammari, Shaimaa K. Mohamed

**Affiliations:** 1 Department of Chemistry, College of Science, Jouf University, Sakaka P.O. Box 2014, Saudi Arabia; aaessawy@ju.edu.sa (A.A.E.); mrelaassar@ju.edu.sa (M.R.E.-A.); 2 Egyptian Petroleum Research Institute, Cairo 11727, Egypt; mohamed_betiha1@hotmail.com; 3 Physics Department, College of Science, Jouf University, Sakaka P.O. Box 2014, Saudi Arabia; ahalshammari@ju.edu.sa; 4 Department of Chemistry, Faculty of Science, Suez University, Suez 8151650, Egypt

**Keywords:** titania nanotubes, Zn (II) ions, visible light, photocatalysis, organic pollutants, rhodamine B

## Abstract

Tailoring high-efficiency photocatalytic composites for various implementations is a major research topic. 1D TNTs-based nanomaterials show promise as a photocatalyst for the remediation of organic pigments in an aqueous solution. Despite this, TiO_2_ (TNTs) is only photoactive in the UV range due to its inherent restriction on absorption of light in the UV range. Herein, we provide a facile recipe to tailor the optical characteristics and photocatalytic activity of TNTs by incorporating Zn (II) ionic species via an ion-exchange approach in an aqueous solution. The inclusion of Zn (II) ions into the TNTs framework expands its absorption of light toward the visible light range, therefore TiO_2_ nanotubes shows the visible-light photo-performance. Activity performance on photocatalytic decontamination of RhB at ambient temperature demonstrates that Zn-TNTs offer considerable boosted catalytic performance compared with untreated tubular TiO_2_ during the illumination of visible light. RhB (10 mg L^−1^) degradation of around 95% was achieved at 120 min. Radical scavenger experiment demonstrated that when electron (e^−^) or holes (h^+^) scavengers are introduced to the photodegradation process, the assessment of decontamination efficacy decreased by 45% and 76%, respectively. This demonstrates a more efficient engagement of the photoexcited electrons over photogenerated holes in the photodegradation mechanism. Furthermore, there seems to be no significant decrease in the activity of the Zn-TNTs after five consecutive runs. As a result, the fabricated Zn-TNTs composite has a high economic potential in the energy and environmental domains.

## 1. Introduction

TiO_2_ has been one of the most researched materials since its commercialization in the early twentieth century due to its exceptional functional characteristics, environmental friendless, and stability [1,2,3]. TiO_2_ performance in several implementations, particularly photocatalysis and heterogeneous catalysis is tightly connected to shape, phase, and crystallite size, which dictate electronic characteristics [2,3,4,5,6]. These textural and morphological properties may be controlled by appropriate choice of fabrication approach and post-treatments, whereas electric characteristics are often fine-toileted through treatment with dopants [7]. 1D semiconducting nanomaterials consist of nanotubes, nanowires, and nanorods and are important in the current nanotechnology [8,9,10,11,12,13]. They have great potential in various applications, such as sensing, conversion of solar energy, photodetectors, batteries, photocatalysis, and light waveguides [14,15,16,17,18,19,20]. TiO_2_ is a photocatalyst which may be utilized for contaminants remediation, hydrogen production from splitting of water and transformation of organic compounds [21,22,23,24]. In contrast to frequently utilized nanoparticles or bulk particles, nanotubular TiO_2_ may give distinct advantages as a photocatalyst due to the following characteristics [25,26]: (i) 1D design allows for both quick and high-distance electron movement, (ii) nanotubular morphology with great surface features is predicted, and (iii) owing to the large length-to-width ratio for 1D TiO_2_ (TNTs), light scattering and absorption may have significant improvement. As a result, exploring the potential uses of these TiO_2_ nanotubular-based nanomaterials as photocatalysts for desired implementations should be of considerable interest. It should be emphasized, however, that TiO_2_ (TNTs) are solely UV-light photo-active due to their absorption in the UV range. Additionally, TiO_2_ nanotubes (TNTs) have a significant cation-exchange capability [27], which is an essential structural characteristic.

1D alkaline titanates are an appealing precursor for the synthesis of 1D tubular TiO_2_ (TNTs) in gram amounts [28]. Moreover, this approach allows for doping at various stages: (i) in situ-doping at the start of the production of alkali titanates [29,30] or (ii) during an ion exchange operation in which the cationic alkaline ions are replaced with dopant ions and/or protons [31]. When it comes to the influence on the morphology of nanomaterials, these two doping procedures are significantly less damaging than certain more recognized approaches, such as high temperature processing. In situ-doping is the best approach for achieving substitutional doping while preserving the shape of one dimensional tubular TiO_2_. This approach has been previously used to synthesize TiO_2_ nanotubes that have been doped with various metal ions [29,30]. However, this method cannot be applied in the basic medium that is used for the development of tubular TiO_2_ because some ions of transition elements produce insoluble hydroxides. Zinc in the oxidation state (II) is an exceedingly desirable dopant for titanate nanomaterials among transition elements. For example, by replacing Ti (IV) substitutional sites, Zn (II) may generate oxygen vacancies, enabling the development of the rutile crystalline phase and thereby considerably altering the durability of the anatase phase [32,33]. Moreover, the inclusion of Zn (II) in the anatase matrix offers a significant influence on the photocatalytic performance. However, since Zn (II) has a different charge than Ti (IV) and its radius is 0.74, which is much bigger compared with Ti (IV) (0.61), substitutional Zn (II) doping is difficult. The decontamination of organic contaminants has offered remarkable promise in the area of photocatalysis [34,35].

Herein, we enhance the light absorption characteristics of tubular TiO_2_ by a facile Zn (II) ion exchange approach and their usage as a photocatalyst in the visible-light range in water remediation under ambient circumstances. TiO_2_ (TNTs) may be converted into a robust visible-light photocatalyst by the introduction of Zn (II). In addition, the Zn (II) doping may boost the performance of TiO_2_ (TNTs) when exposed to visible light due to the increased lifespan of photo-generated e^−^/h^+^ couples.

## 2. Results and Discussion

### Materials Characterization

**Figure 1:**
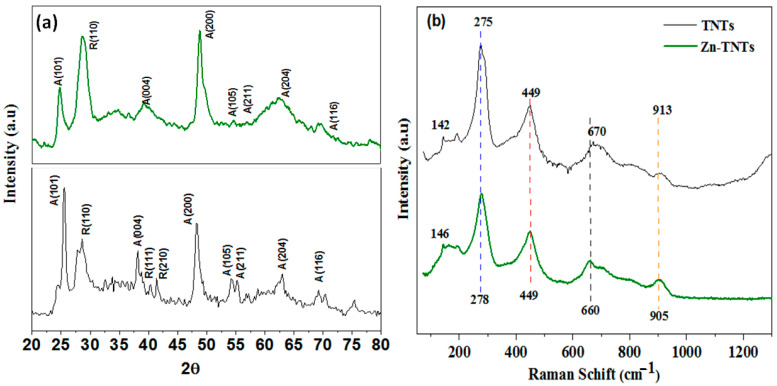
(**a**) XRD patterns and (**b**) Raman spectroscopy of undoped TNTs and Zn-doped TNTs (Zn-TNTs).

The powder X-ray diffraction (XRD) assessment was utilized to establish the tubular nanostructure when preserved in Zn-TNTs as compared with pure TNTs. As depicted in Figure 1a, the diffraction bands at 2θ values of 25.5, 38.3, 48.1, 54.5, 55.4, 63.1, and 69.3° could be indexed to (101), (004), (200), (105), (211), (204) and (116) faces of TNTs anatase structure, respectively. Therefore, this measurement indicates that the prepared TNTs are highly crystalline with anatase structure as a dominant phase (JCDS 21-1272). Additionally, three distinctive rutile diffraction peaks with (110), (111), and (210) faces were also observed (JCDS 21-1276). All of the typical crystalline phase peaks of TNTs were preserved in Zn (II)-ions doped TNTs, indicating that the TNTs had not crumpled. Furthermore, no distinguishable diffraction peaks resembling zinc oxide or hydroxide can be seen in any of the XRD patterns (Figure 1a). Furthermore, regarding un-treated TiO_2_, the conversion to TiO_2_ (anatase) and subsequently to TiO_2_ (rutile) occurs at high temperatures. The existence of Zn (II) ions in the TNTs matrix reduced the conversion of anatase to rutile phase substantially [36,37].

Figure 1b depicts the Raman spectra of the pure TNTs and Zn-TNTs. Tubular nanostructures developed with the formation of two distinctive bands at 275 and 913 cm^−1^ along with a unique rutile mode at 449 cm^−1^. The peak at 449 cm^−1^ corresponds to the phonon (Eg) band of rutile-kind, whereas others are anatase kind. The smallest frequency phonon (Eg) mode of anatase in the Raman spectrum of TNTs changed from 142 cm^−1^ in pure TNTs to 146 cm^−1^ in Zn-TNTs, demonstrating that the tetrahedron architecture was produced in tubular nanostructure. The 4 cm^−1^ blue shift might be due to the deficiency of oxygen. The reorganization of nanomaterials into novel phases is displayed by the disappearance of certain traditional peaks for TiO_2_ (196 cm^−1^) and the emergence of additional wide peaks. The titanate phase is shown by the band at 913 cm^−1^. This peak is related to the symmetric stretching mode of a titanate’s short Ti-O bond in an architecture nanostructure [38].

**Figure 2:**
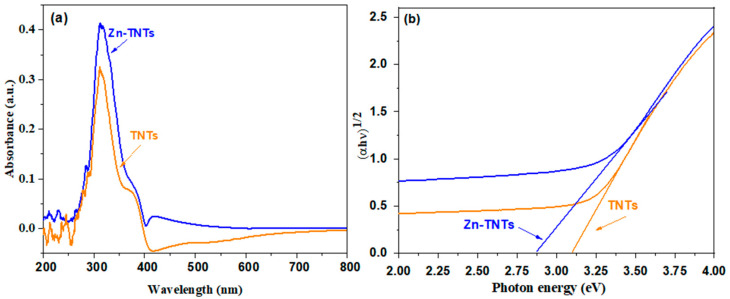
(**a**) UV-vis spectra and (**b**) Tauc’s grphs of undoped TNTs and Zn-doped TNTs (Zn-TNTs).

The optical characteristics using UV-vis of TNTs and Zn-TNTs are depicted in Figure 2, demonstrating that the light absorption property of TiO_2_ nanotubes may be carefully tailored by the addition of various ionic species. The addition of ionic species to the framework of tubular TiO_2_ is committed to expanding its light absorption to the visible-light range. As a result, Zn(II)-ion-treated TNTs may need to be activated by visible light irradiation and are likely to exhibit TNT’s visible light photocatalytic ability for various applications.

A plot obtained via drawing ${\left(\alpha \mathrm{hv}\right)}^{\frac{1}{2}}\;$ against (hν) based on the Tauc’s equation is displayed in Figure 2b, by which the approximately determined bandgap values are 3.12 and 2.873 eV, respectively, for TNTs and Zn-TNTs.. This implies that the bandgap of Zn (II)-ion-treated TNTs lessens when compared with pristine TNTs. This could be attributed to the development of defect energy levels of Zn (II)-ion-treated TNTs between the conduction and valence band of tubular TiO_2_.

**Figure 3:**
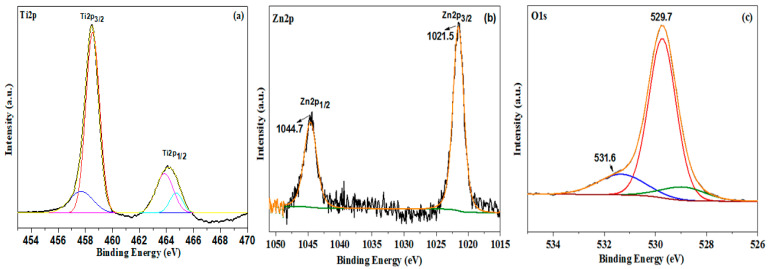
De-convoluted XPS spectra of (**a**) Ti2p, (**b**) Zn2p, and (**c**) O1s of Zn-TNTs nanocomposite.

The electronic properties and chemical oxidation states of Zn (II)-ion-treated TNTs were examined using XPS [39,40,41,42,43]. The high-resolution XPS spectra of Ti2p, Zn2p, and O1s for Zn-TNTs are displayed in Figure 3. The Ti2p_1/2_ and Ti2p_3/2_ bands may be de-convoluted into two pairs of bands, with stronger peaks at 464.1 eV and 458.5 eV relating to Ti^4+^ ions, suggesting the predominant oxidation state of Ti in Zn-TNTs (Figure 3a) [39]. Ti^3+^ is responsible for a set of weaker bands at 457.1 eV and 460.3 eV. This could be attributed to the lower temperature’s treatment: oxygen is insufficiently active to completely oxidize Ti, resulting in the creation of vacancies of O and Ti^3+^ centers in the tubular TiO_2_ (Figure 3a) [40]. On the other hand, the bands centered at 1021.5 and 1044.7 eV in the Zn spectrum (Figure 3b) are related to the Zn 2p_3/2_ and Zn 2p_1/2_ of Zn^2+^, respectively [41]. The concentration of Zn^2+^ in Zn (II)-ion-42treated TNTs is estimated to be 0.48 mol % based on the XPS data quantification. The O1s peak in Figure 3c could be deconvoluted into two bands at 529.7 eV and 531.6 eV. The Ti-O bond is responsible for the peak at 529.8 eV [40]. The presence of a minor band at 531.6 eV might be attributed to oxygen in Ti–O–Zn. Furthermore, no bands for any extraneous atoms were identified, indicating that the fabricated nanocomposite is exceedingly pure, supporting the effective development of the Zn-TNTs nanocomposite.

**Figure 4:**
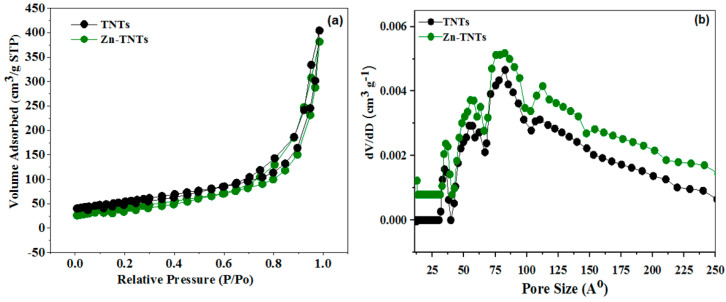
(**a**) N_2_ isotherms (**b**) pore size distribution of undoped TNTs and Zn-doped TNTs (Zn-TNTs).

Nitrogen adsorption–desorption experiments have been utilized to assess the surface characteristics of TNTs and Zn-TNTs. The full isotherms and the associated pore size distributions (BJH) are displayed in Figure 4. Type IV isotherms (Figure 4a) with H3 hysteresis loops were identified in both samples. It is possible that the observed hysteresis in the data presented in Figure 4a is indeed small, which may suggest that the materials have limited porosity. The surface areas of the untreated TNTs and Zn-TNTs were determined to be quite comparable, measuring 235 and 228 m^2^ g^−1^, respectively. Furthermore, they exhibit comparable the same volume of pore of 0.721 and 0.691 cm^3^ g^−1^ for TNTs and Zn-TNTs, respectively. The pore size distribution (Figure 4b) is distinguished by two different wide peaks at 5.6 and 8.3 nm pore width, and a single sharp peak at 3.6 nm. The peaks were allocated to wall flaws of the interior pores of the tubes, and the space between tubes [43]. As a result, the combined BET and XRD results indicate that metal-ion treatment has no influence on the microstructure of Tubular TiO_2_.

**Figure 5:**
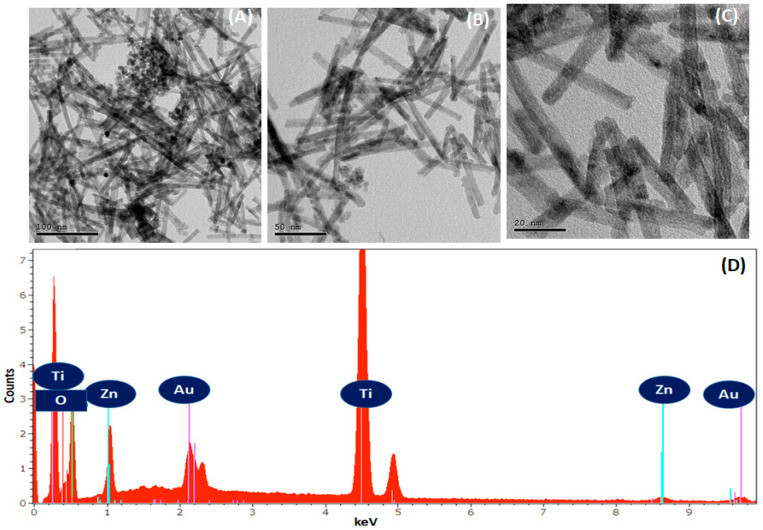
HRTEM with different magnification (**A**–**C**) and EDX analysis of Zn-doped TNTs (Zn-TNTs) (**D**).

High-resolution transmission electron microscopy (HRTEM) images with various magnifications of Zn-TNTs reveal a highly ordered and uniform morphology, as depicted in Figure 5A–C. The nanotubes appear as long, cylindrical structures with a length of several micrometers [44,45]. The elemental assessment of the as-fabricated tubular TiO_2_ and Zn-TNTs systems was explored by energy dispersive X-ray spectrometry. Typical spectra are depicted in (Figure 5D). Titanium, oxygen, and zinc were present by the samples as predicted in the structure of titanate. The weak Au signal is due to the sample coating.

## 3. Adsorption and Photocatalytic Performance

The catalytic activity of as-fabricated TNTs and Zn-TNTs was examined for the remediation of RhB. The finding includes the adsorption of RhB in darkness and the remediation of RhB using the prepared materials under visible illumination. It is evident that there are disparities in the adsorption and degradation performance of RhB using TNTs and Zn-TNTs.

### 3.1. Adsorption Study

To find out the effectiveness of the prepared materials in reducing or removing RhB dye pollutants, the adsorption of the RhB dye on the surfaces of TNTs or Zn-TNTs was studied individually. As can be easily noted, the solution containing RhB was kept in the dark with the prepared materials prior to illuminating it. This was done to attain an equilibrium state of adsorption/desorption and to investigate the adsorption of RhB. The obtained findings for NTs and Zn-TNTs show that uptake equilibrium was attained after 30 min. The high surface area of Zn-TNTs is evidently responsible for their substantial adsorption capacity, particularly towards RhB, as shown in the study. These results indicate that the samples could potentially serve as effective adsorbents for color compounds. The kinetic findings of the uptake of RhB over TNTs and Zn-TNTs were examined utilizing the pseudo-first-order [46] and the pseudo-second-order models [47]. To determine the most suitable kinetic model, the regression factor (R^2^) was used for quantitative evaluation, as presented in Table 1. The findings indicate that the pseudo-first-order model was determined to be the most suitable for describing the adsorption of RhB.

In order to gain a deeper understanding of the adsorption mechanism of RhB onto the synthesized materials, the adsorption data were fitted using commonly used mathematical models for adsorption: the Freundlich Equation (1) and Langmuir Equation (2) models.

LnQ_e_ = LnK_f_ + 1/nLnC_e_ (Freundlich) (1)

Ce/Q_e_ = Ce/Q_max_ + 1/Q_max_K_L_ (Langmuir) (2)

where 1/n and K_F_ are the variables of the Freundlich isotherm. The Langmuir maximum capacity and adsorption constant are represented by Q_max_ and K_L_, respectively. Table 2 displays all the parameter values and regression factors for both isotherms.

It can be inferred from the correlation coefficients of both isotherms that the Langmuir model is the most suitable for describing the adsorption process of RhB. This suggests that the adsorption sites of the prepared materials are homogeneous and that a monolayer of RhB molecules is formed on the surface of these solids.

### 3.2. Photocatalytic Study

**Figure 6:**
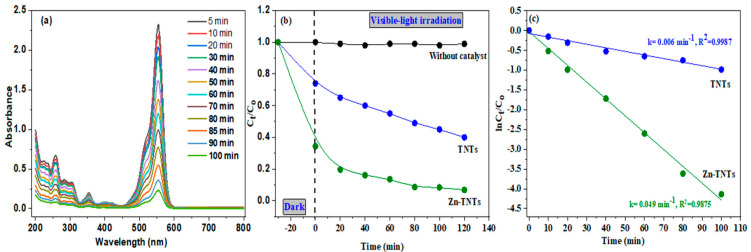
Displays (**a**) the changes in the absorption spectrum of RhB over time during solar-driven photocatalytic degradation in the presence of Zn-TNTs, (**b**) the photocatalytic performance for the degradation of RhB under visible-light irradiation, and (**c**) the kinetic evaluation for RhB degradation utilizing both TNTs and Zn-TNTs.

Figure 6a displays the remediation of RhB using visible illumination for TNTs and Zn-TNTs. Initially, we conducted the decontamination of RhB without TNTs or Zn-TNTs as a control test to gain a better understanding of the remediation process. The results revealed that after 120 min of photolysis, there was little degradability (<2%) of RhB. It demonstrated that the RhB cannot even be adequately remediated using solely photolysis during visible-light irradiation; the degradation (%) of RhB was determined to be 61% for TNTs and 97% for Zn-TNTs after 120 min of visible-light illumination. The UV–vis spectra of RhD in the presence of Zn-TNTs at different time intervals are depicted in Figure 6a. The total photocatalytic efficiency of tubular TiO_2_ during irradiation with visible-light is extremely poor owing to decreased visible-light absorption due to its higher bandgap (3.12 eV), and partially decontaminated RhB for this nanostructure material might be attributed to auto-sensitization. Zn-TNTs outperformed TNTs in photocatalysis efficacy due to their increased absorption in the visible light spectrum (Figure 6b). These findings demonstrated that modifying TNTs with Zn (II) ions might significantly increase their photocatalytic activity during visible-light irradiation.

The decontamination kinetics have been assessed using Langmuir-Hinshelwood model to measure the rate constant for the degradation process [48], utilizing Equation (3):

(3) $$Ln\left(\frac{C_{t}}{C_{0}}\right)=-kt$$

where *C*_0_ and *C_t_* are the initial and ultimate RhB contents, respectively. *k* denotes the rate constant of the pseudo first-order kinetic model, which was estimated for TNTs and Zn-TNTs from the slope of a straight line in the graph between −*Ln*(*C_t_/C*_0_) against time (*t*), as illustrated in Figure 6c. Obviously, both samples display a remarkable linearity as verified by the regression coefficient (R^2^), indicating that the decontamination of RhB obeys pseudo first-order model with K values of TNTs, and Zn-TNTs were 0.006 min^−1^, and 0.049 min^−1^, respectively. By linking the results of RhB dye adsorption and the photocatalytic efficiency, it is clear that the increase in the adsorption efficiency is related to the catalytic efficiency, as the results suggest that the rate of dye photodegradation correlates with the rate of photocatalytic in simulated sunlight. 

To confirm the degradation of the dye, we performed a Chemical Oxygen Demand (COD) experiment, which is a commonly used technique for measuring the content of organic matter in wastewater. The COD measurement test assesses the entire amount of oxygen needed for organic matter oxidation to CO_2_ and H_2_O. A significant decrease in COD levels suggests that the carbon level of the sample has degraded, thereby displaying the extent of mineralization that has taken place. This process causes organic carbon to transform into gaseous CO_2_ [49]. The mineralization process, which involved the opening of the aromatic rings to generate carboxylic acids temporarily, and the production of CO_2_ via the “photo-Kolbe” process, significantly reduced the COD levels (Table 3). The COD reduction indicates that a remarkable amount of mineralization occurred. Based on the significant decline in COD, it’s probable that Zn-TNTs have helped the color molecules entirely mineralize.

### 3.3. Effect of Radical Scavenger

**Figure 7:**
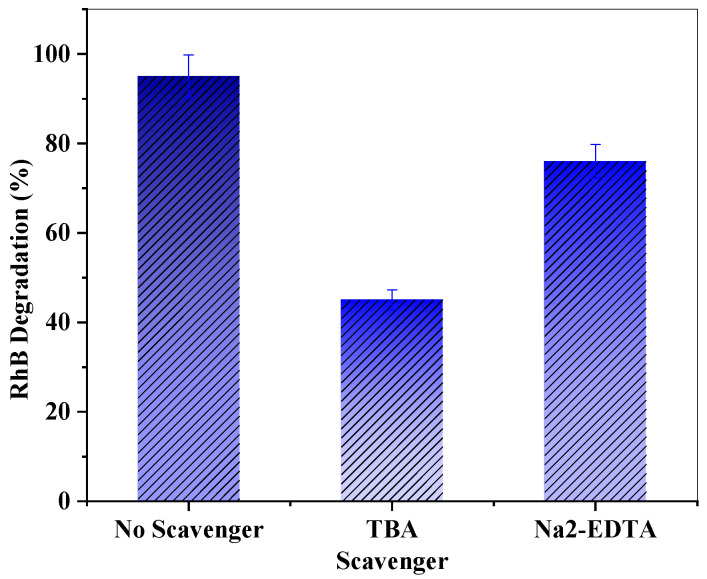
The photodegradation activity of Zn-TNTs in decontamination of RhB under visible-light irradiation in the existence of Na_2_-EDTA as hole scavenger and TBA as electron scavenger.

The trapping experiments were performed in order to recognize the active constituents that were engaged in RhB decontamination, and the results are illustrated in Figure 7. Quenching compounds such as tert-butyl alcohol (TBA) as an electrons (e^−^) scavenger and ethylene-diamine-tetraacetic acid disodium (Na_2_-EDTA) as holes (h^+^) scavenger were utilized throughout the experiments [50,51]. When e^–^ or h^+^ scavengers were introduced to the photodegradation experiment, the assessment of the decontamination efficacy decreased by 45% and 76%, respectively. This demonstrates a more efficient engagement of the photoexcited electrons over photogenerated holes in the photodegradation process.

### 3.4. Stability and Reusability

**Figure 8:**
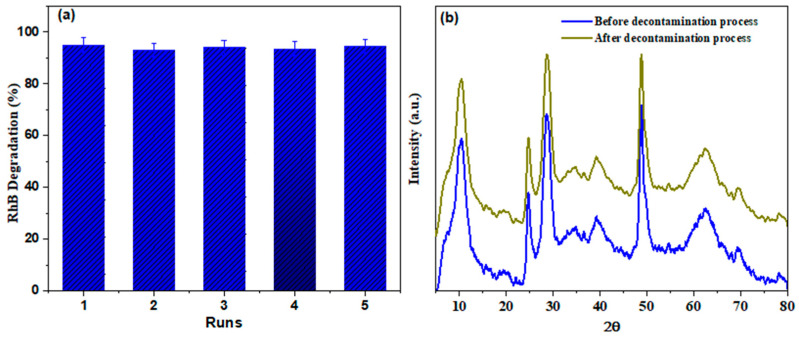
(**a**) Durability of Zn-TNTs in photodegrading RhB up to five runs under visible-light irradiation for 120 min, and (**b**) XRD patterns of Zn-TNTs before and after 5 consecutive runs.

Because photocatalyst durability is a crucial criterion of efficacy assessments for industrial purposes or practical implementations, the reusability of Zn-TNTs photocatalyst was tested utilizing recycling measurement under the similar conditions for RhB degradation. Figure 8a illustrates that there is just no significant decrease in the activity of the photocatalyst after 5 consecutive runs, suggesting that Zn-TNTs composite is stable for decontamination of RhB. Furthermore, the contents of the leaching Zn (II) ions throughout the degradation process. It worth noting that there were no measurable zinc (II) ions during the whole decontamination process. The stability of Zn-TNTs was further assessed by comparing the XRD pattern (Figure 8b) of the Zn-TNTs that is reused for 5 consecutive runs of decontamination of RhB with the comparable fresh Zn-TNTs. As demonstrated, there is no discernible difference in the major XRD pattern before and after decontamination, showing that the tubular structure is stable.

### 3.5. Photocatalytic Mechanism

**Figure 9:**
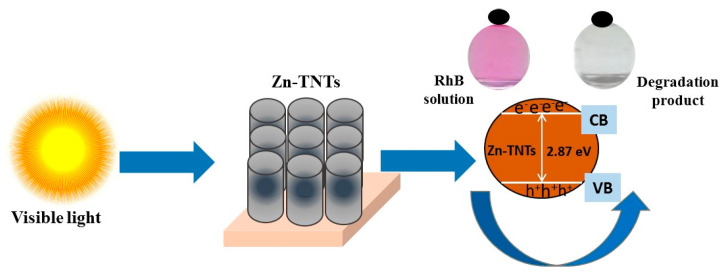
Suggested tentative mechanism for photocatalytic decontamination of RhB over Zn-TNTs.

A hypothetical photocatalytic decontamination mechanism for RhB dye over Zn (II)-treated TNTs could be suggested as the following, which is graphically depicted in Figure 9. When exposed to the visible-light illumination, the Zn (II) ionic species acting as defect energy-levels may boost the absorption of visible light and the development of the photo-excited e^−^/h^+^ couples on of Zn-TNTs surface [52,53,54,55]. The photo-induced electrons were transported to both semiconductors’ conduction band, in which they interact with molecular oxygen to develop O_2_^−^. Simultaneously, photo-excited holes in the valence band of Zn-TNTs interacted with water to form OH• radicals. Following that, as the principle active radicals, these produced radicals engaged in the photocatalytic decontamination of RhB pigment.

## 4. Experimental

### 4.1. Materials

NaOH (≥98%), ethanol (95%), Zinc acetate dehydrate (Zn (CH_3_COO)_2_ 2H_2_O, ≥98%) as zinc-ion precursor, Titanium (IV) oxide (<25 nm, anatase, 99.7%), NH_4_OH solution (28%, ≥99.9%), and rhodamine B (≥95%) were obtained from Sigma-Aldrich, St. Louis, CO, USA. All of the materials are analytic grading and utilized exactly as supplied. The distilled water utilized in all of the studies was obtained from the Milli-Q direct 8 purification system (Millipore, France).

### 4.2. Preparation of Tubular Titanate (TNTs)

TNTs was fabricated by stirring the blend of 1 g titania powder (anatas) and a concentrated solution of NaOH (80 mL, 10 M) to produce a uniform suspension. Afterwards, the material was transferred to a Teflon-lined autoclave and subjected to hydrothermal treatment at 140 °C for 15 h. Following the process, the product was filtrated and rinsed with DI water until it became basic with a PH~ 8. It must be seen that the rinsing operation with a pH of 8 is critical for retaining the complete tubular matrix nanostructure for subsequent heat treatment [36]. Ultrasonication was then used to disperse the obtained precipitate in ethyl alcohol. After a second bath and separation, the final product was vacuum-dried for 12 h at 90 °C. Finally, the final product is calcined at 400 °C in air for 5 h.

### 4.3. Preparation of Zn (II)-Doped Tubular Titanate (Zn-TNTs)

Typically, the zinc (II)-ion precursors of Zn (CH_3_COO) _2_ were completely dissolved in water to develop the appropriate Zn (II) solution, and then a diluted ammonia solution (28%) was added dropwise to generate transparent solutions at ambient temperature. Then, the tubular TiO_2_ (1 g) was suspended in the corresponding Zn (II) solution (100 mL) and agitated for 24 h at a speed of 200 rpm at ambient temperature to enable a suitable ionic-exchange reaction. Finally, the Zn-TNTs products were rinsed many times with ammonia solution (28%), DI water, and a regulation pH of 8 to prevent physisorption of the Zn (II) ions on the nanotubes surface [36].

### 4.4. Characterization

To identify the phase and crystalline nanostructure of the tubular materials, X-ray diffraction (XRD) was employed using an X-ray diffractometer (XPERT, PANalytical, Minneapolis, MN, USA) with Cu K radiation (λ = 0.154 nm) at 40 kV and 40 mA. The diffractograms were obtained in the 2θ range of 20–80°, with a step size of 0.01° and a time interval of 10 s for each step. Raman spectra were obtained using a ProRaman-L instrument (Enwave Optronics, Irvine, CA, USA) with a doubled Nd: YAG laser (λ = 532 nm). X-ray photoelectron spectroscopy (XPS) with Al Kα (200 eV and 50 eV) was performed using equipment from Thermo-Fisher Scientific (K-ALPHA, San Francisco, CA, USA). N_2_ isotherms were measured at 77K using a surface analyzer (NOVA 3200, Quantachrome Instruments, Boynton, FL, USA), and the Barrett–Joyner–Halenda (BJH) model was used to evaluate the pore size distribution from the adsorption branch of the isotherms. Morphology and elemental composition were studied using a high-resolution transmission electron microscopy (HRTEM, JEOL-2011, 200 kV, Tokyo, Japan) equipped with an energy dispersive X-ray (EDX). The optical characteristics were determined using a UV-vis spectrophotometer, Agilent Cary 60 Spectrophotometer (Agilent Technologies, Santa Clara, CA, USA).

The absorption-coefficient α(ν) was evaluated using Equation (4):

(4) $$\alpha (\nu )=\frac{1}{d\;}\log\left(\frac{I_{0}}{I}\right)$$

where *I*_0_ and *I* are the incident and transmitted intensities of light, respectively. E_g_ is the optical band-gap (E_g_) and was determined from the absorption coefficient *α*(ν) utilizing Equation (5)

*αhν* = [*B*(*hν* − *E_g_)*]^*r*^ (5)

where *hν* is the incident light’s photon energy and *B* is the constant of band tailing. For direct or indirect bandgap, *r* is ½ or 2, respectively [37]. The Tauc’s plots were developed by graphing ${\left(\alpha hv\right)}^{\frac{1}{2}}\;$ versus (*hν*) to assess the value of bandgap energy.

### 4.5. Photocatalytic Performance

Typically, in a double wall jacket glass reactor, 50 mg of TNTs or Zn-TNTs were introduced in 100 mL Rhodamine B (RhB) solution with an initial concentration (10 mg L^−1^) to perform a photodegradation experiment at 25 °C. The suspension was then put vertically in front of a visible-light source (Xenon lamp) (300 W, 200 W/m^2^, 460 nm). An aliquot of the reaction mixture was collected out and filtrated using centrifugation at predetermined time intervals. Then the RhB content was then assessed using a UV-vis spectrophotometer (UV-vis spectrophotometer Agilent Cary 60 Spectrophotometer) at 554 nm. Atomic absorption spectroscopy (AAS, iCE3300 AAS atomic absorption spectrophotometer Thermo Fisher, Waltham, MA, USA) was utilized to determine the amount of the leachable Zn(II) ions.

## 5. Conclusions

Herein, 1D TiO_2_ (TNTs) treated with Zn (II) ions was fabricated by an ionic exchange approach in an aquatic medium. The optical characteristics of TNTs are quite-tailored by the inclusion of Zn (II) ions. Specifically, the inclusion of Zn (II) ions into the TNTs framework enables the extension of its light absorption to the visible range, leading TNTs to exhibit visible-light photoactivity toward RhB decontamination. During illumination with visible-light, Zn (II) treated TNTs material exhibits significantly robust catalytic performance toward RhB decontamination compared with the pristine TNTs. There is no significant decrease in the activity of the photocatalyst after 5 consecutive runs, suggesting that Zn-TNTs composite is stable for decontamination of RhB. Obviously, our study shows the tailored optical characteristic of TiO_2_ by treating with Zn (II) ions and, importantly, offers a new avenue of 1D TiO_2_ (TNTs) and other nanostructures as a novel form of visible-light-driven materials for remediation of organic dyes in aquatic environments.

## Figures and Tables

**Table 1:** Kinetics variables for the uptake of RhB using pseudo-first-order, and pseudo-second-order over TNTs and Zn-TNTs.

	Pseudo-First-Order			Pseudo-Second-Order		
Sample	q_e1,cal._ (mg/g)	K_1_ (1/min)	R^2^	q_e2,cal._ (mg/g)	K_2_ (g/mg-min)	R^2^
TNTs	16.24	0.0110	0.9638	16.73	0.0103	0.9591
Zn-TNTs	37.35	0.0364	0.9927	38.34	0.0365	0.9911

**Table 2:** Langmuir and Freundlich isotherm constants for RhB uptake on TNTs and Zn-TNTs.

Sample	Langmuir Isotherm			Freundlich Isotherm		
	q_m,cal._	KL	R^2^	KF	*n*	R^2^
	(mg/g)	(L/mg)		(mg/g)(L/mg)1/n		
TNTs	99	0.251	0.9971	14.1	0.3	0.9321
Zn-TNTs	146	0.342	0.9982	22.7	0.7	0.9732

**Table 3:** Chemical oxygen demand (COD) changes of RhB over the Zn-TNTs at different times.

Physico-Chemical Variables	Pre-Photocatalytic Degradation	In Dark for 60 min	Post-Photocatalytic Degradation after				
			10 min	20 min	40 min	60 min	180 min
COD (ppm)	28.5	18.1	14.6	12.1	8.4	3.1	1.4

## Data Availability

Not applicable.

## Sample Availability

Not available.
